# How to promote, improve and test adherence to scientific evidence in clinical practice

**DOI:** 10.1186/1472-6963-5-62

**Published:** 2005-09-19

**Authors:** Caterina Caminiti, Umberto Scoditti, Francesca Diodati, Rodolfo Passalacqua

**Affiliations:** 1 Epidemiology Service, Azienda Ospedaliero-Universitaria di Parma, Via Gramsci, 14, Parma, Italy; 2 Division of Neurology, Azienda Ospedaliero-Universitaria di Parma, Via Gramsci, 14, Parma, Italy; 3 Division of Medical Oncology, Azienda Ospedaliera di Cremona, Viale Concordia, 1, Cremona, Italy

## Abstract

**Background:**

Negative variation in the management of patients with the same clinical condition is frequent, and affects quality of care. Recent studies indicate that single interventions are not an effective solution. We aim to demonstrate that a multifaceted strategy can favor the introduction of research into practice, and to assess its long-term effects on a set of common medical conditions exhibiting significant negative variation at our institution.

**Methods:**

The strategy, devised and agreed upon by a multidisciplinary group, was first applied to one relevant medical condition – cerebral ischemic stroke. To test its effectiveness a quasi-experimental study was conducted, comparing an intervention group with historical controls. After validation the strategy was extended to other pathologies, and its long-term effect measured using evidence-based quality indicators. Adherence to each indicator was determined prospectively on a six-month basis for a period of at least two consecutive years. Measures are expressed as proportions with 95% confidence intervals.

**Results:**

Validation findings demonstrated that the strategy improved compliance with scientific evidence: the percentage of patients who received a CT scan within 24 hours of hospital presentation rose from 56% to 75%, (χ^2 ^= 7.43 p < 0.01); admissions to selected wards increased from 45% to 64%, (χ^2 ^= 7.81 p < 0.01); the number of physical medicine visits within 24 hours of the request grew from 59% to 91% (χ^2 ^= 14,40 p < 0.001). Over a four-year period the program was gradually applied to 14 medical conditions. Except for 3 cases, compliance with the pathway, i.e. number of eligible patients for whom data on the care process is collected, was above the minimum requirement of 75%. Indicator adherence generally exhibited a positive trend, though variability was observed both among different conditions and between different semesters for the same pathology.

**Conclusion:**

According to our experience, incorporation of research into practice can be favored by systematically applying a shared, multifaceted strategy, involving multidisciplinary teams supported by central coordination. Institutions should device a tailor-made approach, should train personnel on implementation strategies, and create cultural acceptance of change. Just like for experimental trials, human and economic resources should be allocated within health care services to allow the achievement of this objective.

## Background

A large variance in the utilization of health technology (concerning both drugs, equipment, and medical/surgical procedures as well as the organizational and support framework for such performances) is increasingly evident, putting patients at risk of receiving unnecessary care, or of being deprived of performances already proven effective [1-3]. This awareness has made quality of care a central issue [4]. In the past few years various approaches to this phenomenon have been developed, suggesting alternative methodological solutions involving different professional groups. Quality assurance (QA), technology assessment (TA), clinical epidemiology (CE), and continuous quality improvement (CQI), are just a few of the disciplines involved in the critical and constructive evaluation of clinical practice. The main and widely accepted indications suggested by research in this field [5,6] are the following: 1) in order to ensure effectiveness, organizational, socio-cultural, and structural aspects must be taken into account; 2) in order to improve clinical practice the sources of problems must be identified, and all parties must be actively involved; 3) in order to verify and improve the quality of care, accurate local data must be readily available. Following the above-mentioned indications, we have decided to develop and implement at our hospital a multifaceted strategy, comprising the creation and application of integrated care pathways (ICPs), and the monitoring of quality indicators (QIs).

In this article we first describe this strategy and aim to demonstrate that it improves adherence to scientific evidence and consequently reduces negative deviations in patients with stroke. We then describe our experience over a four-year period with the gradual extension of the strategy to other eligible medical conditions and the testing of its long-term effect.

## Methods

The study began in June 2000 and was implemented at one of the largest Italian health care facilities, with 1420 beds (of which 330 for out-patients and 90 for long-term care), and over 60,000 admissions per year.

This initiative was carried out as part of a project sponsored by the Italian Ministry of Health, and was designed in order to provide health planners with indications on factors which favor or hinder the routine use of clinical "best practice", in a natural context of a health care facility, and to suggest the rationale for new intervention studies.

### Strategy's definition and validation

The strategy comprises a sequence of interventions, each designed to remove barriers which have been demonstrated by preceding studies to hinder EBM practice [2]. It was developed with the involvement of various professionals: clinicians, nurses, administrators, epidemiologist, sociologist, patients' representative; this factor has made it possible to devise a shared program, suited to our local reality. Integrated care pathways, quality indicators and relative audit were chosen as tools necessary to health care workers to define the best process in the workplace and monitor variation.

#### ICPs' creation and application

**Figure 1:**
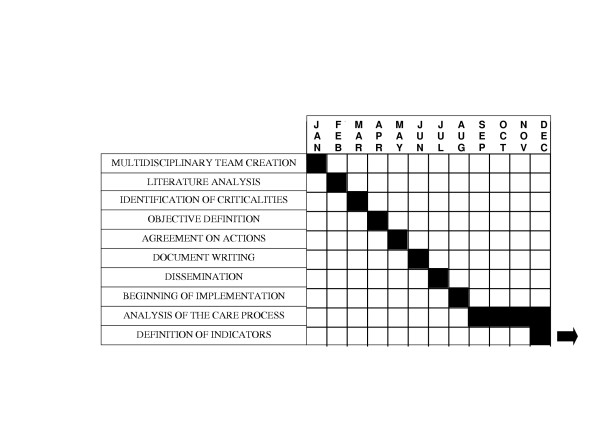
Phases of ICP development and relative mean times.

Integrated care pathways are structured multidisciplinary care plans which detail essential steps in the care of patients with a specific clinical problem [7]. What follows is the description of three main aspects of the process followed for the creation and application of ICPs at our institution, outlined in detail in Figure 1 along with the relative mean times.

##### 1. Multidisciplinary team (MT) creation

Motivated clinicians and nurses involved with the condition join the MT voluntarily. While the MT should not be too large, it is important that all departments involved with the pathology be represented. The members will then appoint the coordinator – a key figure for the success of the pathway.

##### 2. Agreement on objectives and actions

The MT meets regularly to identify key aspects and discuss local problems of the process of care. The team's discussion is based on the evidence gathered through a careful literature analysis. This will eventually lead to the creation of a document which contains the ICP's objectives, required interventions, the description of the pathway through time-task matrices, and the data collection sheet used for the analysis of the care process. This document will be subject to periodical reviews and modified when appropriate. The document is disseminated to all professionals involved with the pathology in question, in order to build consensus and favor its application.

##### 3. Implementation phase

It's a sort of experimental stage, necessary to educate health care professionals on the use of the ICP; during this phase the data, gathered with an ad hoc data collection sheet, will be used to describe the care process, and MT compliance, i.e. the number of eligible patients for whom the data collection sheet is filled out, will be verified; this value is considered acceptable if greater than 75% [7].

#### Development of indicators and analysis through audit

Based on the analysis of the information collected with paper forms, during the implementation phase, and on detected variation, each member of the MT will suggest possible quality indicators. The MT will discuss them and assess them against criteria such as validity (strength of evidence), reliability and feasibility of data collection (they should be easy to retrieve, calculate and interpret), and sensitivity to change [8]. The indicators which best meet the criteria, about 3–6, will be chosen, and form the minimum set of QIs, gathered prospectively using an electronic record [9] and monitored to measure adherence to scientific evidence. To ensure the correct interpretation of observed variations, inclusion and exclusion criteria for each QI are explicitly defined and considered during data processing.

Multiprofessional audit is performed regularly (approximately every 3 months) in order to discuss any negative deviations, to determine the causes, and act upon them if needed [7,9-13]. This should ensure that improvement is gradually achieved and maintained.

#### Study design and statistical analysis for validation

The above-mentioned strategy was first applied to one relevant medical condition – cerebral ischemic stroke – in order to test its effectiveness. We opted for a quasi-experimental design, a controlled study in which an intervention is assigned without the use of randomization [14]. To determine whether the introduction of the intervention had changed behavior in terms of improved adherence to scientific evidence, we compared clinical records of all patients with cerebral ischemic stroke consecutively hospitalized during 2 homogeneous periods, preceding and following the strategy's introduction. The size of the considered sample, about 100 cases per group, was adequate to detect a difference of at least 20%, with significance level α = 0.05 and power 1-β = 0.80. The comparison between proportions was examined using the χ^2 ^test [15]. We considered P values < 0.05 to be significant, P values < 0.01 strongly significant and P values < 0.001 extremely significant. Data were processed using the SAS System (version 8.1).

### Strategy's extension and long-term effects

Following the validation phase, the strategy was gradually applied to other medical conditions chosen according to the following criteria: high admission frequency (at least 100 admissions/year), large variations in clinical practice affecting patient outcome, and high level of interest among local staff. Frequency and variation are verified through the analysis of discharge summaries, clinical records, or studies conducted at a local level for other purposes.

The activity of the MTs is coordinated by the Support Panel (SP). This group – made up of 1 epidemiologist, 1 professional nurse, 1 data manager, and 1 bibliographic researcher – verifies that the chosen medical conditions meet the selection criteria, tests compliance with the program, intervenes in the case of conflict, and provides support to the management in the collection and analysis of data and literature.

For the medical conditions which completed all the steps required by the multifaceted strategy, we recorded a minimum set of quality indicators to monitor the strategy's long-term effect. Data analysis was carried out following the methodology commonly applied in descriptive observational studies [14]. The analysis consisted in the prospective collection of specific indicators, which must be gathered for all eligible patients (following the inclusion criteria established by the multidisciplinary team) consecutively admitted to hospital in a given period (the same dynamic target population). The analysis of such data enables to assess adherence to key aspects of the care process, both in terms of constant improvement and maintenance of improvement overtime once it has been achieved. We determined adherence to each indicator on a six-month basis for a period of at least 2 consecutive years (long-term effect). Each measure is expressed as a proportion with relative 95% confidence interval.

## Results

### Strategy's validation

We compared two periods: the first 6 months of 2001, when the intervention was in use, and the corresponding period of the previous year, when it was not. Trained personnel examined 224 clinical records of eligible patients discharged with a primary diagnosis of cerebral ischemic stroke. The comparison employed the following 3 quality indicators, adapted from the indications established in 1999 by the American Heart Association/American College of Cardiology [16]:

- Performance of a CT scan within 24 hours of hospital presentation;

- Patient admission to dedicated wards;

- Timely involvement of the rehabilitation team

The results of the comparison between the intervention and control groups, carried out to demonstrate improvement of behavior, are the product of the following specific interventions agreed upon by the MT (table 1):

**Table 1:** Pre-post comparison of compliance with QIs for Cerebral Ischemic Stroke

	PRE-INTERVENTION		POST-INTERVENTION	
INDICATOR	N. patients assessed	Value (95%CI)	N. patients assessed	Value (95%CI)

Performance of CT scan within 24 h of hospital presentation	110	56% (47–65)	114	75% ^++ ^(67–83)
Admission to a dedicated ward	110	45% (36–54)	114	64% ^++ ^(55–73)
Early intervention of the rehabilitation team *	57	59% (46–72)	64	91% ^+++ ^(84–98)

Level of test significance χ^2^

+ p < 0.05

+ + p < 0.01

+ + + p < 0.001

* Patients for whom rehabilitation was indicated.

- Cerebral CT scan is crucial to determining the type of stroke and consequently planning the correct therapy [17]. Earlier, patients with suspected stroke usually underwent CT scan in the admission ward, often experiencing important delays, while in some cases the test was not performed. It was therefore decided to provide CT scans for suspected stroke in the emergency department. After the intervention the percentage of patients who received a CT scan within 24 hours of hospital presentation rose from 56% to 75%, (χ^2 ^= 7.43 p < 0.01).

- At our institution, stroke patients used to be hospitalized according to bed availability, which resulted in high dispersion. Considering the great importance of specialized stroke care, as demonstrated in the literature [18], the ICP requires that all subjects presenting to the ER with suspected stroke be reported by telephone to one of the referring physicians who will be in charge of the patient. Consequently, admissions of stroke patients to selected wards increased from 45% to 64%, (χ^2 ^= 7.81 p < 0.01).

- Prior to the intervention, physical medicine evaluation and rehabilitation were not always promptly initiated, mostly due to a lack of communication between the wards and the physical medicine department. Since early intervention is central in improving disability and quality of life [18], a referring physical medicine physician was chosen who is immediately informed via fax when a new stroke patient is admitted to hospital. This will enable the rapid provision of an individualized evaluation and rehabilitation program. After the introduction of this measure the number of patients with rehabilitative indication seen by the rehabilitation team within 24 hours grew from 59% to 91% (χ^2 ^= 14,40 p < 0.001).

### Strategy's extension and long-term effects

Over a four-year period the strategy was gradually applied to 14 medical conditions, involving nearly 150 health care workers, and allowing the hospital management of over 7000 patients/year to be verified.

The 14 conditions have reached different stages, depending on the time they were included in the program, and also on factors related to the individual MTs. Table 2 provides for each pathway initiation date, MT composition, number of eligible patients/year, and MT compliance. The latter has been recorded for 13 out of 14 ICPs – in June 2004 the one on Pulmonary Thromboembolism had yet to begin implementation. Except for the ICPs on Breast Cancer, Supraventricular Tachiarrhythmias, and Pediatric Head Injury (53%, 55%, and 62% respectively), compliance was good, reaching optimal rates for Liver Cirrhosis (94%), Stroke (89%), Pediatric Pneumonia and Melanoma (88%), Child Delivery (86%), and chronic obstructive pulmonary disease (COPD) (84%).

**Table 2:** Integrated care pathways in use at our institution

ICP	Initiation Date	Eligible/Year+	MT Composition	MT Compliance ++
Cerebral Ischemic Stroke	June 2000	**220–230**	2 emergency medicine physicians, 1 geriatrist, 1 physical medicine physician, 1 neurologist, 1 neuroradiologist, 2 internists, 1 psychiatrist, 1 physiotherapist, 2 nurses.	**89%**
Pediatric Head Injury	June 2001	**100–110**	1 neuropsychiatrist, 2 pediatricians, 1 radiologist, 2 nurses.	**62%**
Chest Pain	June 2001	**1800–2000**	3 cardiologists, 1 cardiosurgeon, 2 emergency medicine physicians, 2 internists, 3 nurses.	**79%**
COPD	June 2001	**430–450**	3 pulmonologists, 1 emergency medicine physician, 1 geriatrist, 1 physical medicine physician, 2 nurses.	**84%**
Child Delivery	June 2001	**2000–2100**	3 gynecologists, 1 pediatrician, 1 obstetrician, 1 nurse.	**86%**
Lung Cancer	June 2001*	**200–220**	3 pulmonologists, 2 thoracic surgeons, 1 oncologist, 1 radiologist, 1 radiotherapist, 1 physical medicine physician, 1 pathologist, 2 nurses.	**77%**
Breast Cancer	December 2001	**320–340**	2 oncologists, 3 surgeons, 1 plastic surgeon, 1 radiologist, 1 radiotherapist, 1 pathologist, 1 biologist, 1 nurse.	**53%**
Pediatric Pneumonia	December 2001	**110–130**	6 pediatricians, 1 neonatologist, 1 radiologist, 2 nurses.	**88%**
Liver Cirrhosis	December 2001	**470–490**	2 gastroenterologists, 3 infective disease physicians, 2 nurses.	**94%**
Supraventricular Tachiarrhythmias	March 2002	**680–700**	3 cardiologists, 4 internists, 2 emergency medicine physicians, 1 nurse.	**55%**
Hip Arthroplasty	October 2002	**360–380**	4 orthopedists, 1 radiologist, 1 physical medicine physician, 1 anesthetist, 1 hematologist, 2 nurses.	**82%**
Melanoma	January 2003	**110–130**	3 dermatologists, 2 plastic surgeons, 1 nuclear medicine physician, 2 oncologists, 1 general surgeon, 1 pathologist, 1 nurse.	**88%**
Non-Hodgkin Lymphomas	June 2003	**90–110**	1 oncologist, 1 radiotherapist, 1 radiologist, 2 hematologist, 1 nuclear medicine physician, 1 surgeon, 2 internists, 1 nurse	**77%**
Pulmonary thromboembolism	April 2004	**190–210**	1 emergency medicine physician, 2 pulmonologists, 1 nuclear medicine physician, 2 internists, 1 geriatrist, 1 cardiologist, 1 radiologist	*

* For this pathway the implementation phase was not completed by June 2004, thus data are not available.

+ Eligible/Year: number of patients who meet the inclusion criteria set forth in the ICP document.

++ MT Compliance: number of eligible patients for whom the data collection sheet is filled out; this value is considered acceptable if greater than **75%**.

By June 2004, indicators had been defined for 7 conditions. For 5 of those (Stroke, Chest Pain, COPD, Child Delivery, Lung Cancer) the effect of the strategy has been monitored for at least 2 years; for the remaining two (Liver Cirrhosis and Pediatric Pneumonia) indicators have been monitored since the second semester of 2003, and thus are not included in our long-term analysis. For three conditions (Supraventricular Tachiarrhythmias, Breast Cancer and Pediatric Head Injury) the minimum set of indicators had not been measured. Hip Arthroplasty, Melanoma and Non-Hodgkin Lymphomas had completed their implementation phase, and the MTs were working on defining the quality indicators to begin monitoring.

Table 3 shows the trend of adherence to each indicator for five semesters, the two semesters of 2002 and 2003, and the first semester of 2004, except for Lung Cancer for which only 4 semesters are shown because implementation was delayed.

**Table 3:** Trend of adherence to each indicator by semester

CONDITION	INDICATOR	1^st ^semester 2002 Percentage (95%CI)	2^nd ^semester 2002 Percentage (95%CI)	1^st ^semester 2003 Percentage (95%CI)	2^nd ^semester 2003 Percentage (95%CI)	1^st ^semester 2004 Percentage (95%CI)
STROKE	CT scan performed within 24 hours of hospital presentation[17]	**91%** *(86–97)*	**90%** *(84–96)*	**96%** *(92–100)*	**96%** *(93–99)*	**93%** *(88–98)*
	Patients admitted to a dedicated ward[18]	**63%** *(56–71)*	**74%** *(67–81)*	**81%** *(75–87)*	**80%** *(74–86)*	**82%** *(75–89)*
	Visits by the physical medicine specialist within 24 hours of request for patients with rehabilitative indication [18]	**78%** *(69–87)*	**65%** *(55–75)*	**94%** *(89–99)*	**82%** *(74–90)*	**75%** *(66–84)*
	Patients transferred to a post-acute care facility according to their clinical indications [41]	**89%** *(78–100)*	**78%** *(64–91)*	**80%** *(72–89)*	**79%** *(72–86)*	**73%** *(64–82)*
	Psychiatric tests (GDS) administered to patients eligible for the test [42]	**67%** (58–78)	*57%* **(46–69)**	**73%** (64–83)	**74%** *(65–83)*	**75%** *(66–84)*
	Patients discharged alive who underwent 1 follow-up visit 1–3 months after the onset of stroke [16]	**57%** *(47–68)*	*67%* (57–76)	**80%** *(72–88)*	**69%** *(60–78)*	**74%** *(65–83)*
CHEST PAIN	Patients discharged from the ED and readmitted to hospital within 1 month [43]	**3.2%** *(1–6)*	**1.4%** *(0–3)*	**0.5%** *(0–2)*	**1.9%** *(0–4)*	**0%**
	Hospitalized patients admitted to the appropriate ward according to pain characteristics [44]	**64%** *(61–67)*	**61%** *(58–64)*	**81%** *(78–84)*	**73%** *(70–76)*	**77%** *(74–80)*
	Patients with AMI admitted to the coronary ICU within 60 minutes of hospital presentation [45]	**63%** *(55–70)*	**65%** *(58–72)*	**77%** *(70–84)*	**70%** *(63–77)*	**76%** *(69–83)*
	Diagnoses of AMI in the discharge summaries inconsistent with clinical records [46]	**8%** *(5–11)*	**9%** *(5–13)*	**7%** *(4–10)*	**7%** *(4–10)*	**5%** *(2–8)*
COPD	Patients hospitalized in the appropriate ward according to severity [47]	**64%** *(57–71)*	**62%** *(55–69)*	**75%** *(69–81)*	**92%** *(88–96)*	**86%** *(81–91)*
	Patients eligible for spirometry who underwent spirometry [48]	**56%** *(49–63)*	**49%** *(41–56)*	**92%** *(88–96)*	**92%** *(88–96)*	**96%** *(93–99)*
	Patients with MRC dyspnea grade >= 3 on admission who improved on discharge [49]	**86%** *(79–92)*	**83%** *(77–90)*	**91%** *(86–96)*	**88%** *(82–94)*	**87%** *(81–93)*
	Patients hospitalized for the appropriate number of days according to clinical severity [50]	**93%** *(88–98)*	**98%** *(95–100)*	**96%** *(93–100)*	**94%** *(89–99)*	**97%** *(94–100)*
CHILD DELIVERY	Cesarean sections on the total number of child deliveries [51]	**41%** *(38–44)*	**41%** *(39–44)*	**35%** *(32–38)*	**39%** *(36–42)*	**39%** *(36–42)*
	Elective cesarean sections for women with previous cesarean section without contraindications to vaginal delivery [52]	**66%** *(57–75)*	**60%** *(51–69)*	**45%** *(36–54)*	**64%** *(55–73)*	**56%** *(47–65)*
	Cesarean sections performed because of the woman's psychological refusal of vaginal delivery [53]	**25%** *(18–32)*	**17%** *(11–23)*	**18%** *(12–24)*	**15%** *(10–20)*	**20%** *(15–25)*
LUNG CANCER	Patients included in the ICP within 2 weeks of the first X-ray [54]		**53%** *(43–62)*	**55%** *(43–67)*	**61%** *(51–71)*	**62%** *(51–73)*
	Patients diagnosed within 4 weeks since ICP inclusion [54]		**73%** *(64–82)*	**73%** *(63–83)*	**78%** *(69–87)*	**62%** *(51–73)*
	Staged patients [55]		**89%** *(83–95)*	**97%** *(93–100)*	**93%** *(88–98)*	**96%** *(92–100)*
	Patients transferred to the intensive care unit for the first 24 hours after surgery [56]		**93%** *(87–99)*	**87%** *(77–97)*	**81%** *(70–92)*	**86%** *(76–96)*
	Patients who underwent surgery according to eligibility criteria [55]		**91%** *(84–98)*	**97%** *(93–100)*	**88%** *(81–95)*	**91%** *(85–97)*
	Borderline cases discussed by the multidisciplinary group [55]		**100%**	**100%**	**100%**	**100%**

Some indicators exhibit extremely high adherence, constant through time; for instance, percentage of borderline lung cancer cases discussed by the multidisciplinary team, performance of a CT scan in stroke patients, and appropriate length of stay (LOS) for COPD patients according to severity, have never fallen below 90% in the observed semesters.

Other indicators began with a low, or fairly low adherence and then exhibited considerable improvements; for instance, the percentage of eligible patients receiving spirometry rose from 56% to 96%. The same positive trend was observed for indicators relating to the appropriateness of admission: stroke patients admitted to a dedicated ward (63%–82%); patients with chest pain hospitalized in the appropriate ward according to pain characteristics (64%–77%); and COPD patients hospitalized in the appropriate ward according to severity (64%–86%).

Finally, for some indicators adherence rates are still low compared to the standards found in the literature; for example, the percentage of cesarean sections (CSs) (39% in the last semester) remains far above the WHO recommendation of a 15% rate. Analogously, the percentage of lung cancer patients included in the ICP within 2 weeks of the first X-ray, on which suspicion is based, remains fairly poor in the 4 analyzed semesters.

**Figure 2:**
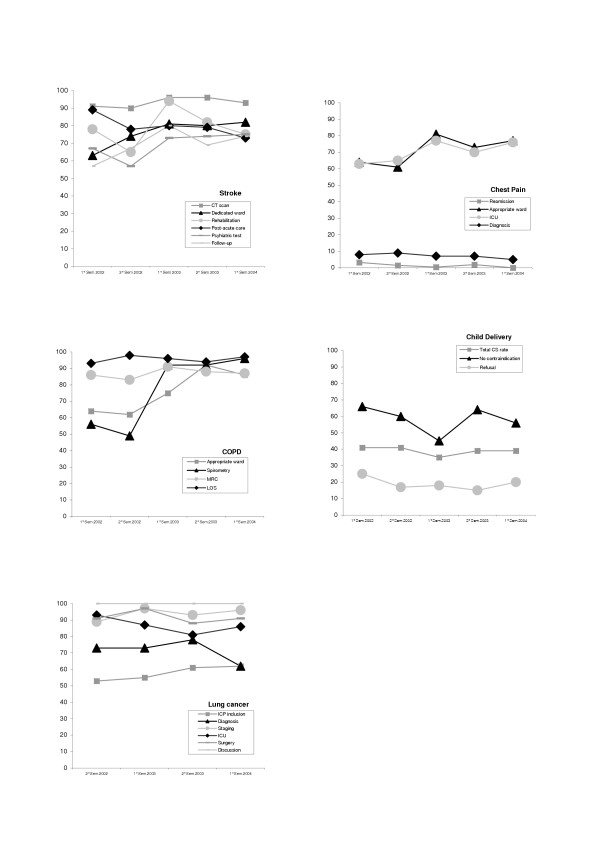
**Trend of adherence to each indicator by semester**. For each condition the proportion of patients receiving key interventions after the introduction of the multifaceted strategy is displayed. See text for the full description of indicators and for bibliographic references.

For further clarity, trends for five medical conditions are portrayed graphically in Figure 2.

## Discussion

In order to improve adherence to evidence-based medicine at our hospital, we applied a planned strategy comprising a combination of interventions, each addressing specific barriers to change (from professional, socio-cultural, and organizational viewpoints); we have demonstrated through a quasi-experimental study the effectiveness of this strategy. The improvement trend we demonstrate is consistent with the findings reported by Kwan et al in a very recent before-after study on the application of a Stroke pathway, concerning the indicators on timely CT scan performance and early involvement of the rehabilitation team [19].

The execution of the program we propose is complex (it requires meetings, debates, data collection, etc.), but so is the process of incorporation of evidence into practice; in fact, despite publication of several management guidelines in virtually any medical field, the literature shows that adherence to EBM indications is often poor [2,20-22]. The biggest obstacle seems to be the perception of guidelines as tools distant from the reality in which one operates, and the large amount of information often contained in guidelines, which clinicians have no time to digest [20,23]. The picture is complicated by the gap between those who practice and those who manage health care, whereby the introduction of new technologies/tests and the implementation of change is sometimes left to the initiative of individual professionals, or, conversely, allows administrators to activate or cancel procedures without assessing the effects of their decisions at a local level with those directly involved (health care workers and users). We tried solving these problems by creating a constant debate between administrators and health care workers, from the initial stages of the improvement strategy's definition.

We have demonstrated the strategy's capability to improve adherence to scientific evidence through a quasi-experimental study with historical controls, and not a randomized trial. RCTs are the gold standard for testing effectiveness, however in our case this design would have posed a number of methodological issues. For complex interventions aiming to change behavior, like ours, randomization by individual patients is not feasible and cluster randomization, by doctor or practice, is usually advised [24]. This approach, however, would have been extremely complex to take in our study, because the strategy in question involves multiple organizational levels (Emergency Department, Internal Medicine wards, Neurology, Rehabilitation, Radiology service). Furthermore, since the intervention's effectiveness depends on the subjects' active participation and on their choices, random allocation may lessen its effect, and a clinical trial would in fact be inappropriate [25,26].

The analysis of clinical records as a means of validating the program also has some well-known limitations (exhaustivity, accuracy, etc.), however it offers the great advantage of allowing timely access to the information needed to documenting an intervention's impact using a historical control. A possible bias may exist in the interpretation of the measured effect, which may be due to greater accuracy of documentation rather than to a real change in care. However, since a pathway's success is strictly related to the quality and quantity of available information on the patients' characteristics and the tests they undergo, the appropriate management of clinical information is part of our work's objective.

Our work concerns the adoption of an improvement strategy in a large hospital involving a set of common medical conditions, and its long-term effectiveness. Studies on the subject generally focus on a single condition, or on a few easy-to-standardized pathologies [27-34]. Instead, our work concerns the use of 14 ICPs, of which 77% exhibit good compliance with the strategy. Noteworthy is also the number of patients, approximately 7000/year, whose hospital management is verified through: the analysis of data prospectively collected, the comparison with the most recent scientific indications, and the discussion of deviations by a group of experts directly involved in the management of the condition. What makes our experience different is that it focuses on the strategy's effectiveness in the long-run, while research on ICPs found in the literature generally reports on short-term findings. A very recent Australian study [35] does focus on the long-term effect of ICP use, but the number of patients included in the analysis is quite small.

Despite some variability, data generally demonstrate a positive trend, showing improvements for most indicators with fairly frequent drops in both second semesters, which most probably reflect the greater difficulties encountered during the summer holiday season, when personnel is reduced, thus affecting the provision of services. The measures we adopted to achieve change in behavior were both educational (critical literature analysis, sharing and dissemination of the ICP, systematic clinical audits, etc.), and organizational/managerial (performance of CT scans in the emergency department, rapid involvement of the rehabilitation team via fax, use of a portable spirometer for bedridden patients, etc.). As also noted by Panella et al in a recent Italian experience with the application of an ICP program [36], constant dialogue within MTs and between managers and clinicians, based on variance analysis, is essential to ensure best clinical practice.

The employment of this strategy is not easy, and some medical conditions have encountered greater difficulties than others. The ICP on Breast Cancer has been inactive since September 2002; though it was initiated in December 2001, it has not yet completed its implementation phase, and indicators have not yet been defined. This pathway's problems are mostly caused by the instability consequent to the change of the director of the Oncology Department, which led to the lack of a leading figure in the multidisciplinary group, an essential aspect for improvement of clinical practice [7]. The ICP on Pediatric Head Injury was also inactive since January 2002. Indicators have been defined but never applied. The main obstacle for this ICP seems to be the concern of many physicians of losing their professional freedom, also fearing possible ethical and legal implications in the care of children [37]. The ICP on Supraventricular tachiarrhythmias, which was in its implementation phase, shows the lowest MT compliance, 55%. The difficulty seems to lie mainly in the large amount of data to be collected, since the MT had not identified the most interesting key aspects, and in the fact that this group of patients is managed in several different wards. Finally, the ICP on Child Delivery has proven very ineffective, exhibiting very low adherence with the selected QIs. Various major obstacles have been identified for this ICP: first of all, the lack of sound scientific evidence supporting the pathway's indications, which were derived from authoritative sources (WHO) but are mostly based on expert opinion and remain controversial. Furthermore, it is believed that the high and rising cesarean section rates in most countries may be due in part to physician attitudes of defensive medicine. Legal suits are very frequent when a vaginal delivery has a bad outcome, but they are unlikely when an unnecessary cesarean section is performed [38]. Protection against legal litigation would thus be required to enhance compliance with indications aiming to reduce unnecessary cesarean section rates. Other actions which could help achieve WHO goals are the provision of epidural anesthesia free of charge (in most Italian institutions it is rather costly for patients), and the equalization of financial compensation for CS and vaginal delivery, since in Italy the former is much higher and this might encourage some gynecologists to support the performance of unnecessary CSs.

## Conclusion

This research highlights the importance of multidisciplinary involvement in the creation and application of a strategy to favor EBM introduction into practice. We believe hospitals should ensure the incorporation of research into routine practice by systematically applying a shared, multifaceted strategy, as single interventions cannot be effective in all situations [2]. Our experience confirms what recently emphasized in the literature, i.e. that multiple approaches are the most effective, and that the choice of interventions should be guided by the analysis of determinants of professional behavior and by specific clinical and organizational circumstances [39]. Health care institutions willing to adopt analogous tools should first assess local characteristics carefully and develop a tailor-made approach.

The presence of central coordination, competent in scientific methodology and implementation techniques, is necessary to support the work of individual multidisciplinary teams, to favor agreement between health care professionals and managers, and to constantly remind all parties involved of the importance of the program, as motivation is likely to decrease with time. A far-reaching change in culture is essential to favor the acceptance of change in an organization. Institutions should offer their professionals adequate training on implementation strategies and on the nature and importance of clinical practice improvement [40].

The main down side of this program, as emphasized in the literature on quality improvement strategies [7] is its high costs in terms of staff time. This can be a cause of reluctance for some clinicians and managers. However, the introduction of scientific evidence into practice does necessarily require time and money. We therefore believe that, just like for experimental trials, economic and professional resources should be allocated to allow the achievement of this objective.

## Competing interests

The author(s) declare that they have no competing interests.

## Authors' contributions

CC is the person responsible for the program discussed in the paper. She conceived of the study, and supervised the entire process, from the creation of the program to its application, to statistical analysis, to the drafting of the manuscript. US is the coordinator of the MT on cerebral ischemic stroke. He worked on the definition of the pathway and was responsible for the data gathering process. FD performed bibliographic research and literature analysis, participated in the coordination of the work, and helped to draft and translate the manuscript into English. RP offered expertise on the methodology to follow, and provided comments and suggestions in the preparation of the paper. All authors read and approved the final manuscript.

## Pre-publication history

The pre-publication history for this paper can be accessed here:


